# Intravascular Large B-Cell Lymphoma: A Diagnostic Dilemma

**DOI:** 10.7759/cureus.16459

**Published:** 2021-07-18

**Authors:** Arya Mariam Roy, Yadav Pandey, Derek Middleton, Brannon Broadfoot, Appalanaidu Sasapu, Muthu Veeraputhiran

**Affiliations:** 1 Department of Internal Medicine, University of Arkansas for Medical Sciences, Little Rock, USA; 2 Department of Hematology and Oncology, University of Arkansas for Medical Sciences, Little Rock, USA; 3 Department of Pathology, University of Arkansas for Medical Sciences, Little Rock, USA; 4 Department of Hematology and Medical Oncology, University of Arkansas for Medical Sciences, Little Rock, USA

**Keywords:** intravascular large b-cell lymphoma, b-symptoms, intravascular lymphoma, diffuse large b cell lymphoma, fever of unknown origin, rituximab, bone marrow biopsy

## Abstract

Intravascular large B-cell lymphoma is a rare malignancy characterized by the presence of lymphoma cells within the lumen of blood vessels. The annual incidence of cases is fewer than 0.5 cases per 1,000,000. It usually affects the elderly with an average age of diagnosis around 70 years. Due to the absence of lymphoma cells in the peripheral smear and lymphadenopathy, it is difficult to diagnose these cases. Although the central nervous system and skin are the commonly involved organs, they can involve any organ system. Prompt diagnosis and initiation of treatment are very crucial as it carries a high mortality. We describe two patients who presented with constitutional symptoms and fever of unknown origin, later diagnosed as intravascular large B- cell lymphoma. The diagnosis was difficult in both cases as the presenting symptoms were atypical. One of the patients was diagnosed at autopsy. The delay in diagnosis often leads to fatal outcomes as the disease is very aggressive. A high degree of clinical suspicion is the key to prompt diagnosis and improved outcomes.

## Introduction

Intravascular large B-cell lymphoma (IVLCL) is an extremely rare malignancy characterized by the proliferation of lymphoma cells within the lumen of small blood vessels without an obvious extravascular tumor mass or circulating lymphoma cells [1,2]. Although T- cell and natural killer (NK) cell phenotypes of intravascular lymphoma have been reported [3,4], intravascular large cell lymphoma is considered a subtype of diffuse large B-cell lymphoma in recent WHO classification [5]. The estimated annual incidence of IVLCL is fewer than 0.5 cases per 1,000,000 [6]. Due to its rarity, most of our knowledge on IVLCL comes from case reports and case series. It is an aggressive and fatal malignancy with poor outcomes that usually affects the elderly, with a reported median age of diagnosis around 70 years and with no gender predilection. The central nervous system (CNS) and skin involvement are commonly reported; however, it can involve any organ system in the body including lungs, spleen, and bone marrow [2,7]. Due to its heterogeneous presentation, the absence of lymphoma cells in the peripheral smear, and lack of lymphadenopathy it is often laborious to diagnose the condition timely and most cases will end up recognized only at autopsy. Here, we describe two cases who presented with fever of unknown origin and constitutional symptoms and were diagnosed with IVLCL after a long array of clinical, laboratory, and radiological exams.

## Case presentation

Case 1

A 60-year-old previously healthy African American man presented with a four-month history of dry cough, malaise, intermittent fever, night sweats, and unintentional weight loss. The patient was febrile, ill-looking, pale, and icteric on examination. Initial blood work-up showed anemia, elevated bilirubin, and liver enzymes (Table 1).

**Table 1:** Clinical and laboratory findings at admission WBC - White blood cells; AST - Aspartate aminotransferase; ALT - Alanine transaminase; LDH - Lactate dehydrogenase

Variables	Case 1	Case 2
Fever	Present	Present
B-Symptoms	Yes - fever, weight loss, night sweats	Yes - fever, weight loss,
Hemoglobin (g/dL)	8.6	10.9
WBC (/microL)	4,900	7,400
Platelets (/microL)	213,000	160,000
Bilirubin (mg/dL)	2.3	1.2
AST (IU/L)	121	27
ALT (IU/L)	39	11
LDH (U/L)	542	1,425
Uric acid (mg/dL)	8.7	11.8
Serum sodium (mmol/L)	126	137
Serum potassium (mmol/L)	4.1	3.6
Urea (mg/dL)	5	24
Creatinine (mg/dL)	0.6	1.4
Lactic acid (mmol/L)	1.6	7.8
Lymphadenopathy	No	No
Splenomegaly	Yes	Yes

**Figure 1:**
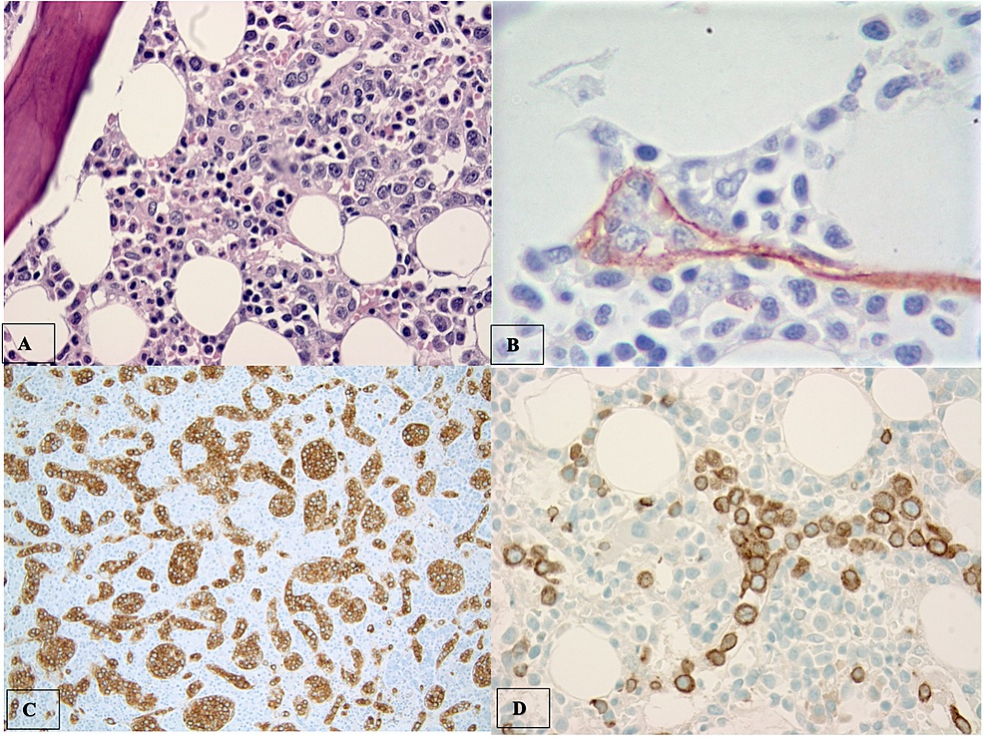
Bone marrow biopsy revealing intravascular large B-cell lymphoma Histologic sections of the bone marrow demonstrated focal aggregates of large, centroblast-like mononuclear cells with irregular nuclei and prominent nucleoli. They were distributed predominantly in a sinusoidal pattern (A – H&E stain, 60x). A CD34 stain highlighted tumor cells which were localized to the small vessels (B – 100x). The atypical lymphoid cells expressed CD20 (C – 20x) and CD79a (D – 60x). Overall, these features were most consistent with an intravascular large B-cell lymphoma.

Peripheral smear showed left-shifted granulocytes, normocytic anemia with no schistocytes, normal platelet morphology. The infectious work-up including bacterial and fungal cultures, viral panel, tick-borne illness serology, and HIV serology was negative. Computed tomography (CT) scan of the chest and abdomen was unremarkable except for splenomegaly. The anemia continued to worsen requiring blood transfusion with new thrombocytopenia and worsening liver function. Bone marrow examination was performed for worsening anemia, fever of unknown origin, and splenomegaly. The bone marrow exam revealed IVLCL (Figures 1A-1D).

The patient received rituximab combined with gemcitabine and oxaliplatin along with a dose of intrathecal methotrexate for CNS prophylaxis. The hospital course was complicated by tumor lysis syndrome and sepsis. The clinical course continued to deteriorate, and the patient opted for comfort care and passed away in the hospital.

Case 2

**Figure 2:**
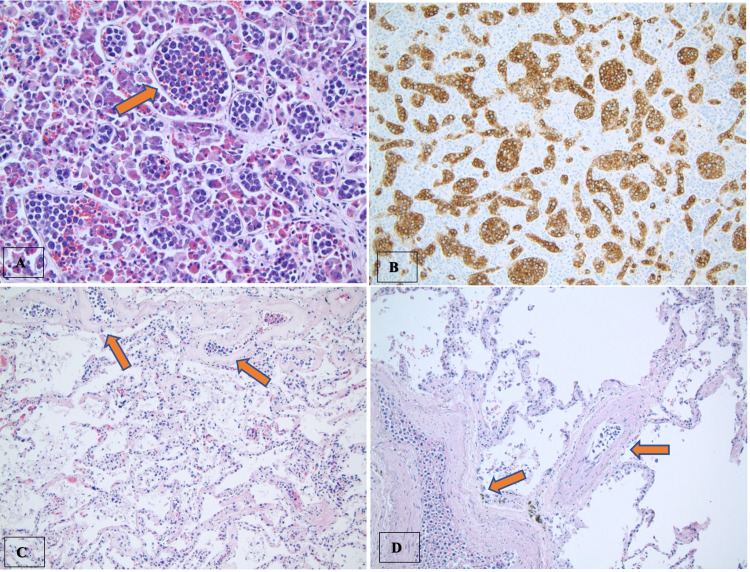
Autopsy findings showing the involvement of IVLCL cells Pituitary gland: The atypical hematolymphoid cells in the blood vessels show large nuclei, open chromatin pattern, prominent nucleoli, and mitotic activity (A, arrow indicate lymphoma cells within blood vessels). These cells are highlighted with the B-cell marker CD20 (B). Lungs: There are numerous large atypical cells present in the vasculature (arrows) that range from capillaries to small and medium-sized vessels. Atypical cell involvement is present also in subpleural vessels and almost all capillaries in the alveolar septae (C, D). IVLCL - Intravascular large B-cell lymphoma

A 71-year-old Caucasian woman was admitted for a two-month history of worsening shortness of breath, dry cough, fatigue, and weight loss. Her past medical history was significant for bronchial asthma, poliomyelitis, hypertension, and diabetes. Family history showed lung cancer in the father and breast cancer in the mother. She was afebrile and was hypoxic with an arterial oxygen saturation of 60% on 4L oxygen supplementation. On initial evaluation, the patient was found to have autoimmune hemolytic anemia and worsening lactic acidosis without clear etiology (Table 1). The patient underwent extensive workup including thyroid hormone levels, antinuclear antibody (ANA) panel, serum immunoglobulin levels, mycoplasma pneumonia antibodies, which were all normal. Peripheral smear showed anemia with anisocytosis, no schistocytes, and thrombocytopenia. CT scan of the abdomen showed mild splenomegaly and bilateral adrenal hyperplasia. She was started on steroids with minimal clinical improvement. Bone marrow biopsy showed hypercellularity (50%) with erythroid predominance. Positron emission tomography-computed tomography (PET-CT) of the vertex to feet was unremarkable. Her respiratory status continued to worsen, and she deteriorated despite supportive treatment. The patient pursued comfort care and passed away in the hospital. The autopsy showed extensive involvement of IVLCL cells within small vascular spaces of all her organs including the brain, heart, skin, lungs, intestine, bone marrow, and her endocrine glands thyroid, pancreas, pituitary, adrenals (Figures 2A-2D).

## Discussion

IVLCL is an aggressive subtype of diffuse large B-cell lymphoma that is often difficult to diagnose and has a poor prognosis. It is characterized by the presence of large lymphoma cells within the lumen of small blood vessels. The absence of cell surface proteins CD29 (B1 integrin) and CD54 (ICAM-1) helps with the diapedesis across the endothelium and the aberrant expression of CD11a and CXCR3 proteins confine lymphoma cells in the intravascular space [2,8]. Based on the geographical origin of the patient, the clinical presentation varies. “Classical variant,” mainly reported in Europeans, is characterized by CNS and/or cutaneous involvement whereas “Asian variant,” commonly reported in Asian decedents is characterized by hemophagocytic syndrome, bone marrow involvement, fever, hepatosplenomegaly, and/or thrombocytopenia [7,9]. Although the overall prognosis is poor, it was reported that the “cutaneous variant,” which has disease solely limited to the skin was found to have significantly better outcomes with treatment [10].

IVLCL can involve any organ system in the body. A meta-analysis by Fonkem et al. found that CNS is the most involved organ (60%) followed by bone marrow and spleen (11%), skin (8%), and lungs (7%). The involvement of the kidney, adrenal glands, reproductive organs, thyroid glands, the gall bladder is also reported [6,11-13]. Clinical presentation can vary based on the organ system involvement. Most of the cases present with constitutional symptoms, fever of unknown origin, B-symptoms, low blood counts, a broad spectrum of neurological diseases ranging from confusion to stroke [14,15] similar to our two patients. Diagnosis is commonly obtained by the biopsy of the involved organ. Even though intravascular involvement is the characteristic finding in IVLCL, peripheral blood involvement is very rarely seen. Bone marrow biopsy can yield diagnosis if bone marrow involvement is present, but sometimes the findings can be nonspecific as in the second case. Laboratory findings usually reveal anemia, thrombocytopenia, and elevated serum LDH. Abdominal imaging should be considered as it commonly involves the spleen and adrenal involvement is also reported in 60% of cases in autopsy. Ground glass opacities can be seen in chest imaging in lung involvement [1,16].

The presentation of our cases was different from the expected geographical variants of IVLCL. Both cases were clinically similar with the Asian variant, but neither of them was Asian decedents. The first patient presented with a fever of unknown origin and had anemia, elevated LDH and liver enzymes, and splenomegaly. Bone marrow biopsy was diagnostic in that case. The presence of severe liver dysfunction precluded the use of doxorubicin and vincristine. He was treated with rituximab, gemcitabine, and oxaliplatin. In the second case, the patient was not started on therapy as the diagnosis was not found even after extensive workup. Bone marrow biopsy was non-diagnostic in that case even though bone marrow involvement was found on autopsy.

Most of the patients with IVLCL presents with disseminated disease or stage IV. Staging workup should include peripheral blood smear, magnetic resonance imaging (MRI) of the brain, bone marrow biopsy, involved organ biopsy, CT scan, or whole-body PET scan besides routine blood workup. Histopathology examination shows large neoplastic lymphoid cells with large nuclei and scant cytoplasm in the blood vessel lumen. Immunophenotypically, besides the strong expression of CD20, it expresses CD79a, MUM1/IRF4, CD5, Pax-5 [1,8].

IVLCL is very aggressive and difficult to treat as it progresses rapidly except for the cutaneous variant. The most commonly used regimen is rituximab along with cyclophosphamide, doxorubicin, vincristine, and prednisone (R- CHOP) which has shown a 88% complete remission rate and three-year overall survival (OS) of 75%-80% in western patients [17]. Rituximab monotherapy can be given in patients with poor functional status. Patients who received autologous hematopoietic stem cell transplantation (auto- HSCT) as consolidation have a prolonged survival rate with a three-year OS of 91%-100% [18]. CNS prophylaxis should be considered as CNS relapse and extravascular CNS dissemination has been commonly reported. Age < 70 years, non-CNS site of initial presentation, LDH < 700, rituximab treatment can be adopted as good prognostic factors [2].

## Conclusions

IVLCL should be considered a differential diagnosis in patients presenting with constitutional symptoms, fever of unknown origin, especially with cytopenia’s, splenomegaly, and elevated LDH. Our cases suggest that a high degree of clinical suspicion is the key to prompt diagnosis as even bone marrow biopsy may fail to reveal the diagnosis and patients can present out of the pictured geographical variants. Timely treatment should be commenced as the disease progress rapidly and can be potentially cured with chemotherapy and stem cell transplant.
